# Psychiatry during the Covid-19 pandemic: a survey on mental health departments in Italy

**DOI:** 10.1186/s12888-020-02997-z

**Published:** 2020-12-16

**Authors:** Bernardo Carpiniello, Massimo Tusconi, Enrico Zanalda, Guido Di Sciascio, Massimo Di Giannantonio, The Executive Committee of The Italian Society of Psychiatry, Enrico Zanalda, Massimo Di Giannantonio, Bernardo Carpiniello, Claudio Mencacci, Matteo Balestrieri, Emi Bondi, Salvatore Varia, Antonio Vita, Guido Di Sciascio, Moreno De Rossi, Mario Amore, Antonello Bellomo, Paola Calò, Giancarlo Cerveri, Giulio Corrivetti, Giuseppe Ducci, Andrea Fagiolini, Bruno Forti, Lucio Ghio, Pierluigi Politi, Paola Rocca, Rita Roncone, Vincenzo Villari, Rocco Zoccali

**Affiliations:** 1 Department of Medical Sciences and Public Health-Unit of Psychiatry, University of Cagliari, Cagliari, Italy; 2 Department of Mental Health, ASL-TO3, Turin, Italy; 3 Department of Mental Health, ASL, Bari, Italy; 4 Department of Neurosciences, Imaging and Clinical Sciences, University of Chieti, Chieti, Italy

**Keywords:** Mental Health Departments, psychiatric assistance, functioning, activities, restrictions, response, Covid-19, Pandemic, Emergency, Italy

## Abstract

**Background:**

To date, very few nationwide studies addressing the way in which mental health services are addressing the current pandemics have been published. The present paper reports data obtained from a survey relating to the Italian mental health system conducted during the first phase of the Covid-19 epidemic.

**Methods:**

Two online questionnaires regarding Community Mental Health Centres (CMHC) and General Hospital Psychiatric Wards (GHPW), respectively, were sent to the Heads of all Italian Mental Health Departments (MHDs). Statistical analysis was carried out by means of Chi Square test with Yates correction or the Fisher Exact test, as needed.

**Results:**

Seventy-one (52.9%) of the 134 MHDs and 107 (32.6%) of the 318 GHPWs returned completed questionnaires. Less than 20% of CMHCs were closed and approx. 25% had introduced restricted access hours. A substantial change in the standard mode of operation in CMHCs was reported with only urgent psychiatric interventions, compulsory treatments and consultations for imprisoned people continuing unchanged. All other activities had been reduced to some extent. Remote contacts with users had been set up in about 75% of cases. Cases of COVID positivity were reported for both staff members (approx. 50% of CHMCs) and service users (52% of CHMCs). 20% of CMHCs reported cases of increased aggressiveness or violence among community patients, although only 8.6% relating to severe cases. Significant problems emerged with regard to the availability of personal protective equipment (PPE) for staff members. A reduced number of GHPWs (− 12%), beds (approx.-30%) and admissions were registered (87% of GHPWs). An increase in compulsory admissions and the rate of violence towards self or others among inpatients was reported by 8% of GHPWs. Patient swabs were carried out in 50% of GHPWs. 60% of GHPWs registered the admission to general COVID-19 Units of symptomatic COVID+ non-severe psychiatric patients whilst COVID+ severe psychiatric patients who were non-collaborative were admitted to specifically set up “COVID-19” GHPWs or to isolated areas of the wards purposely adapted for the scope.

**Conclusions:**

The pandemic has led to a drastic reduction in levels of care, which may produce a severe impact on the mental health of the population in relation to the consequences of the expected economic crisis and of the second ongoing wave of the pandemic.

**Supplementary Information:**

The online version contains supplementary material available at 10.1186/s12888-020-02997-z.

## Background

The COVID-19 epidemic, initially confined to China, has rapidly evolved into a pandemic. Italy was the first Western country to be severely affected by the COVID-19 pandemic. Due to the need to check the spread of the COVID-19 infection, a series of measures were set up by Governments virtually worldwide (i.e. voluntary or compulsory home confinement, restriction on gatherings of large groups of people, cancellation of all public events and a series of domestic and international travel restrictions) [1]. On 31 January 2020, the Italian Government declared a Public Health Emergency of International Concern, and since February 2020 a series of decrees have been progressively issued, ultimately culminating on 21 March in a nationwide lockdown, which lasted until 18 May 2020. At the start of the epidemic, Italy was one of the most heavily affected countries in the World. Indeed, official data provided by the Italian Higher Institute of Health (Istituto Superiore di Sanità) on 17 April 2020 reported 159,107 confirmed cases throughout the country and 19,996 deaths, with an overall fatality rate of 12.6%; the average age of the deceased was 80 years (62 years among infected subjects) with a prevalence of males (almost 70% of deaths); the number of confirmed cases of COVID-19 amongst healthcare professionals amounted to 16,991 (males = 31.9%; average age 48 years) [2]. At the time, Italy was one of the countries with the highest number of infections and deaths worldwide [3]. Although in July Italy was already emerging from the critical phase of the epidemic, the numbers remained impressive: on 7 July 2020 the country reported 241,819 confirmed cases and 34,869 deaths [4]. The epidemic has undoubtedly placed a huge strain on the National Health System, giving rise to grave concerns as to the ability of the system to effectively respond to the needs of infected patients, particularly those requiring intensive care [5]. The impact of the COVID-19 outbreak on mental health is expected to be huge [6–9] and likely long-lasting, thus presenting a series of global challenges that will need to be appropriately addressed [10]. Few nationwide studies relating to the response of mental health services to the current pandemic have been published so far, with the majority of contributions coming from China, the first country to experience an outbreak of the new virus [11–14]. Given the wide disparity present in the organisation of psychiatric services worldwide, knowledge of how an extensive community mental health system such as that present throughout Italy has faced the emergency would be of interest. The transition from a hospital-centred to a community mental health centre care system was first implemented in Italy through a reform dated 1978 [15]. This transition was definitively completed with the closure of Forensic Psychiatric Hospitals which were replaced by small scale therapeutic facilities (Residenze per la Esecuzione della Misura di Sicurezza (REMS) established by Laws 09/2012 and 81/2014 [16]. More than 40 years later, Italian psychiatry continues to rely on a nationwide community care system, although featuring a marked variation in the level and quality of services provided throughout the different regions of the country [17] and the presence of ongoing controversy [18], also with regard to future trends [19]. Although the overall balance of the Reform has been largely positive, a series of challenges to the system continue to arise due, on the one hand, to an excessive burden on mental health services caused by a progressive increase in the number of patients followed and to a diversification in needs of care, whilst on the other from a progressive lack of resources. Moreover, although no trend has been manifested towards re-institutionalisation, a series of concerns have been raised with regard to the overall quality of care in some parts of Italy [17]. Irrespective of whether or not the Italian psychiatric reform may be considered a milestone, it undoubtedly represents one of the most radical attempts to overcome the practice of custodial psychiatry [20], although, due to an economic and cultural crisis, Italy seems to have lost its creativity as well as an interest in mental health, which has been progressively neglected [21]. To date, only local reports relating to Italian psychiatric services have been published [22–26], in addition to a letter from our group focused on the first national data [27]. Indeed, in this post-acute phase, the Italian healthcare system is having to face the challenges posed by the prevailing pandemic. In this report, we present the first set of data obtained from a survey conducted during the acute phase of the epidemic and lockdown by the Italian Society of Psychiatry to assess the impact of the current emergency on the activities of the Italian Mental Health Departments (MHDs), multi-professional units comprising Community Mental Health Centres (CMHCs), Residential Facilities (RFs) and Psychiatric Wards in General Hospitals (GHPWs). According to the latest Mental Health Report issued by the Ministry of Health [28], Italy has a total of 134 active MHDs with 1481 CMHCs, 2346 RFs and 318 GHPWs.

## Methods

Based on the list of Mental Health Departments updated annually by the Italian Society of Psychiatry, between 1 and 11 April 2020, all Heads of MHDs were informed of the aims of the study and invited to take part in the survey conducted by means of a 40-item multiple choice online questionnaire sent to CMHCs and a 30-item questionnaire to GHPWs. The questionnaires were purposely developed for this survey on behalf of the Italian Society of Psychiatry. An English version of both questionnaires may be found as Additional file 1 and Additional file 2. Each questionnaire took 15–20 min to complete. Each respondent was asked to answer all items of the questionnaires based on the available data and/or information they had been privy to as Head of Department. A reminder was sent to all participants between 1 and 11 April 2020. Responses were analysed according to acknowledged geographical macro-areas of Italy (Northern Italy; Central Italy; Southern Italy, including the Islands) and to rates of COVID-19 cases in the reference area for each MHD. For this purpose, rates of cases per 1000 inhabitants were calculated for each Italian region on the basis of data relating to confirmed cases issued by the Italian Ministry of Health on 11 April 2020. Based on the finding of an average national rate of 2.02 × 1000 inhabitants (range 0.42–5.12), two groups were considered as follows: group 1 = ≥2 cases × 1000 (high rate regions); group 2: < 2 cases × 1.000 (medium-low rate regions). Statistical analysis of nominal data was carried out by means of Chi Square test with Yates correction or Fisher Exact test, as needed.

## Results

Seventy-one (52.9%) of the 134 MHDs, and 107 (32.6%) of the 318 GHPWs returned completed questionnaires. 62 out of 134 MHDs (46.3%) are located in Northern Italy (Regions: Piedmont, Val d’Aosta, Liguria, Lombardy, Veneto, Friuli, and Autonomous Provinces of Trento and Bolzano), 36 (26.9%) in Central Italy (Regions: Emilia-Romagna, Tuscany, Umbria, Lazio, Marche, Abruzzo, Molise) and 36 (26.9%) in Southern Italy (Regions: Campania, Puglia, Basilicata, Calabria, Sicily, Sardinia). 71 questionnaires have been returned from the 134 MHDs (52.9% of the total): 33 (53.1%) out of 66 MHDs located in Northern Italy (respondent range by Region: 20–100%), 18 (50%) out of 36 located in Central Italy (respondent range per Region 27.3–100%) and 21 (58.8%) located in Southern Italy (respondent range per Region:0–80%). The difference between respondent and non-respondent MHDs according to geographic distribution is not statistically significant (chi-square test = 0.5147, *p*-value .773112). 107 questionnaires relating to the 318 GHPWs have been returned (33.6% of the total): 49 (37.1%) out of 132 GHPWs located in Northern Italy (respondent range by Region: 22.8–100%), 28 (31.1%) out of 90 in Central Italy (respondent range per Region 8–83.3%) and 35 (26.0%) located in Southern Italy (respondent range per Region:0–57.1%). Likewise, the difference between respondent and non-respondent GHPWs according to geographic distribution is statistically non-significant (chi-square test = 3.1851, *p*-value = .203407).

The main general findings obtained for currently operating CMCHs are shown in Table 1. As a premise, it should be mentioned that these approx. 1400 CMCHs are spread throughout the country and are generally open 5–7 days a week, 12 h per day, in line with regional regulations, with a few units located in specific areas remaining open 24/7. Our data reveal how since the lockdown approx. 13% of these facilities have been closed, and 25% have reduced their hours of access. Moreover, a noticeable decrease (approx. -80%) has been registered in active Day Hospitals (DHs), the semi-residential facilities within MHDs largely involved in clinical monitoring and treatment of subacute, not severe cases. An even greater reduction (− 85%) has been observed in the number of operational Day Centres (DCs), semi-residential facilities focussing on psychosocial and rehabilitation activities. Only Residential Facilities (RFs), the small, (generally 20-bed) units specifically deputed to middle-long term rehabilitation, have remained almost fully operational, although with restrictions in new admissions and discharges. Data relating to ongoing levels and types of activity in CMHCs during this period of emergency are reported in Table 2.

**Table 1:** Community Mental Health Centres and other community facilities operating during the Covid-19 epidemic in Italy

Items	N (%) ofrespondents Yes	Statistically significant differencesaccording to geographical area^**1**^	Differences according to groupsbased upon rates of Covid + cases per 1000 inhabitants on aregional basis^**2**^
CMHCs with regular daily opening	61 (85.9)	None	None
CHMCs with regular daily hours of opening	53 (73.2)	None	None
CMCHs Day Hospital active	16 (22.5)	None	None
CMHCs’ Day Centre active	11 (15.5)	None	None
Residential facilities (RFs) active^3^	71 (100)	None	None

^1^ Italy’s Geographical areas are: N=North, C=Centre, S=South and Islands

^2^ group 1 ≥ 2 cases × 1000 inhabitants; group 2 < 2 cases × 1000 inhabitants;high rate regions are those included in group 1, as above; Low rate regions are those included in class 2 as above

^3^ RFs are small units (20 beds on average) generally managed by private cooperatives or associations, which operate under Regional Health Authority accreditation and the control and supervision of MHDs

**Table 2:** Community Mental Health Centres activities during the Covid-19 epidemic in Italy

Items	N (%) ofrespondents Yes	Statistically significantdifferences accordingto geographical area^**a**^	Differences according togroups based upon ratesof Covid + cases per 1000inhabitants on a regionalbasis^**b**^
Written protocols relating to management ofactivities during the emergency	50 (70.4)	None	High rate regions^c^ = 35(85.4) low rate regions^d^ = 14 (48.3) *p*<.05
Scheduled on-site psychiatric visits^c^	63 (88.7)	None	None
Scheduled at home psychiatric visits^c^	59 (83.0)	None	None
Urgent psychiatric visits on site	71 (100)	None	None
Urgent psychiatric visits at home	68 (95.7)	None	None
Compulsory Treatments	71 (100)	None	None
Individual Psychotherapy^c^	33 (46.4)	none	High rate regions^c^ = 23 (57.4) low rate regions^d^ = 10 (34.4)* p*<.05
Group Psychotherapy^c^	4 (5.6)	None	None
Psychosocial Interventions^c^	1 (1.4)	None	None
Contacts with patients^d^	54 (76.0)	None	None
Scheduled contacts^e^	66 (93.0)	*N* = 40 (100) C = 10 (100)S = 15 (70) *p*<.01	None
Phone and/or Video Counselling for generalpopulation	60 (84.5)	None	None
Phone and/or Video Counselling for healthservices operators	66 (92.0)	*N* = 40 (100) C = 10 (100)S = 16 (76.2) *p*<.01	None
Psychiatric Consultations for General Hospitals^f^	55 (77.4)	None	None
Staff Meetings^g^	56 (78.9)	None	None
Scheduled Administration of LAI antipsychotics	Both on site and athome = 65 (91,5) On site only = 5 (7.0)	None	None
Regular psychiatric monitoring of cases at RFs	32 (46.5)%	*N* = 17 (42.5) C = 5 (50.0)S = 12 (57.1) *p*<.05	High rate regions^c^ = 32 (76.0) low rate regions^d^ = 17 (58.6) *p* < .05
New admissions in RFs suspended	55 (77.4)	*N* = 27 (67.5) C = 9 (90)S = 19 (90,5)* p*<.05	None
Discharge from RFs suspended	59 (83.0)	None	None
Regular Monitoring of offenders entrusted toCMHCs	43 (60.5)	*N* = 32 (80), C = 5 (50),S = 7 (33.3) *p*<.005	None
Psychiatric Consultations in Jails	51 (71.8)	None	None
Reduction of Hospital Admissions	60 (84.5)	None	None
Reduction of Interventions for compulsoryadmissions	47 (66.2)	None	None
Increase of aggressivity toward self or others	15 (21.1)	None	None

^a^Italy’s Geographical areas are: N=North, C=Centre, S=South and Islands

^b^group 1 ≥ 2 cases × 1000 inhabitants; group 2 < 2 cases × 1000 inhabitants;high rate regions are those included in group 1, as above; Low rate regions are those included in class 2 as above

^c^Only in selected cases, when deemed necessary

^d^Contacts were established in case of suspension of on-site or home visits (type of contacts:telephone calls 100%, video calls 67%; e-mails 19%; all types 41%)

^e^Contact scheduled on a regular basis according to individual needs

^f^Psychiatric consultations are delivered by CHCM psychiatrists when the General Hospital in the catchment area is not equipped with a GHPW

^g^On site or in videoconference

Generally, the operational mode in these units is regulated by specific written protocols, particularly in areas featuring higher rates of contagion. Urgent psychiatric consultations, both on-site and at home, are proceeding as usual, in the same way as interventions for compulsory treatments, psychiatric prison consultations and both on-site and home administration of Long Acting Injectable (LAI) antipsychotics. Almost everywhere MHDs have set up remote counselling activities, usually by phone, both for the general population and specifically targeting health workers. All other activities have been affected by a significant decrease. Indeed, scheduled psychiatric consultations, both at home and on-site, have only continued for selected cases, being replaced by scheduled remote contacts, mainly through phone calls with staff members (100% of cases). Video calls have been adopted in 67% of cases and e-mails in 19%, with only 41% of CMHCs using all means of contact. Once the need for a face-to-face consultation was identified during remote contacts, the individual was invited to attend an on-site appointment in compliance with all enforced safety measures (i.e. no accompanying person unless deemed strictly necessary, social distancing of a least 1 meter or more in waiting rooms and in consultation rooms. On accessing the facility patients were asked to fill in a form relating to their personal health status and their body temperature was checked using infrared thermometers; surgical masks were compulsory for both patients and doctors/nurses). Where necessary, patients and/or carers have been provided with surgical masks if available, or invited to return after having procured them. To this regard, it should be noted that the lack of protective masks represented a huge problem throughout Italy during the acute phase of the pandemic; indeed, to address the shortage of masks, the government included in Decree-Law No. 18 of 17 March 2020 (Cura Italia) a new simplified procedure for the validation of new devices, including those designed and developed using 3D printing [29]. No on-site or home appointments were permitted in the presence of suspected infection of patients. When infections were suspected, in line with a specific protocol set up by the Italian Ministry of Health, a report was sent to the Local Crisis Unit involved in managing these cases. In general, subjects forwarded to swab testing were those displaying signs or symptoms of a suspected Covid-19 infection (i.e. fever, cold, cough, dyspnoea, conjunctivitis) and/or who had recently been in close contact with persons having confirmed or suspected infection, or had recently returned from one of the countries severely affected by the epidemic, such as China. Indeed, the shortage of swabs worldwide and supply issues had led the Ministry of Health to emanate a directive advocating the use of tests only in selected populations [30]. All other operations have been affected by a significant decrease, including psychiatric consultations for General Hospitals (approx. -25%), individual psychotherapies (approx. -65%), group psychotherapies and psychosocial interventions (approx. -90/95%), and monitoring of both subjects admitted to Residential Facilities (− 60%) and offenders affected by mental disorders assigned by the Courts to CMCHs (− 45%). As a general rule, staff meetings are going ahead as planned, wherever possible using videoconferencing facilities. Table 3 provides details of safety data relating to CHCMs workers and users.

**Table 3:** Staff and users safety at Community Mental Health Centres during Covid-19 epidemics in Italy

Items	N (%) of respondents Yes	Statistically significant differencesaccording to geographical area^**a**^	Differences according to classesbased upon rates of Covid + cases per 1000 inhabitants on aregional basis^**b**^
Staff concerns about personal safety	65 (91.5)	None	None
PPE (Personal Protective Equipment) available	Thermometers 19 (26.7) Surg. Masks 65 (91.5) FFP2/3 Masks 27 (38.0) Gloves 58 (81.7) Glasses 22 (30.9) Dispos. glows 41 (57.7)	None	None
PPE Evaluation	Adequate = 9 (12.7) Partly adeq = 44 (61.9) Inadequate = 15 (21.1)	*N* = 6 (15.0) C = 3 (30.0) S = 0 (0.0) *p*<.05 *N* = 27 (67.5) C = 6 (60.0) S = 12 (57.0) *not significant* *N* = 5 (12.5) C = 0.0 S = 100*p*<.005	High rate regions^2^ = 5 (11.9) low rate regions^2^ = 10 (34.5) *p*<.001
Reported Covid+ cases among staff members	37 (52.1)	*N* = 33 (82.5) C = 2 (20) S = 1 (4.7)* p*<.0001	High rate regions^2^ = 34 (80.9) low rate regions^2^ = 13 (44.8) *p*<.0001
Reported Staff members in quarantine	47 (66.2)	*N* = 34 (85.0) C = 5 (50.0) S = 7 (33.3) *p*<.001	High rate regions^2^ = 34 (80.9) low rate regions^2^ = 13 (44.8) *p*<.001
Reported Covid+ cases among CMHCpatients	37 (52.1)	*N* = 29 (72.5) C = 3 (30.0) S = 4 (19.0) *p*<.001	High rate regions^2^ = 32 (76.1) low rate regions^2^ = 6 (20.7) *p*<.001
Reported Covid+ cases among RF patients	26 (36.6)	*N* = 23 (57.5) C = 2 (20) S = 0 (0.0) *p*<.0001	High rate regions^2^ = 24 (57.1) low rate regions^2^ = 1 (3.5) *p*<.0001

^a^Italy’s Geographical areas are: *N* North, *C* Centre, *S* South and Islands

^b^group 1 ≥ 2 cases × 1000 inhabitants; group 2 < 2 cases × 1000 inhabitants;high rate regions are those included in group 1, as above; Low rate regions are those included in class 2 as above

The majority of staff members have expressed safety concerns. Major issues in the supply of Personal Protective Equipment (PPE) have been reported for infrared thermometers, high protection masks, safety glasses and disposable gloves. Although no difference in supplies were detected based on macro-areas, a referred lack of equipment was largely registered in southern Italy, namely, the areas featuring prevalently lower rates of infection. COVID+ cases have been reported frequently by both CMHC staff (reported by 52% of MHDs) and facility users (reported by 52% of MHDs), while lower rates have been referred for patients living in RFs. As expected, a significantly higher number of cases have been reported in regions in Northern Italy, the areas featuring the highest rates of infection. Finally, a limited number of MHDs (21.4%) have reported cases of increased aggressiveness or violence, either towards self or others, among community patients, with a mere 8.6% constituting severe cases. General information relating to GHPWs operating during the current pandemic and the activities carried out are shown in Table 4. GHPWs are the public hospital units devoted both to voluntary and compulsory admissions. The process of involuntary admission in Italy is regulated by Law 833/1978, which essentially states that compulsory hospital admission may be proposed by any public or private physician only when three criteria have been met: the person needs urgent treatment; the person refuses the proposed treatment; the required treatments can only be undertaken in a hospital setting. A second certificate issued by a public physician (generally but not necessarily a staff psychiatrist in the MHD) is mandatory in order to obtain a Hospital Treatment Order signed by the mayor of the town, as local Health Authority. The decision of the mayor is submitted to the scrutiny of the Tutelary Judge of the local Court and the patient, his / her family members or anyone who deems hospitalization illegitimate may file an appeal. Compulsory admission may last no more than 7 days, but may be prolonged for additional 1 week periods by the Mayor on request of the head of the GHPW to which the patient has been admitted.

**Table 4:** General Hospital Psychiatric Wards operating and activities undertaken during the Covid-19 epidemic in Italy

Items	N (%) of respondents Yes	Statistically significantdifferences according togeographical area^**1**^	Differences according to classesbased upon rates of Covid + cases per 1000 inhabitants on aregional basis^**2**^
PWs closed	15 (13.1)	None	None
Number of beds reduced	34 (31.8)	None	None
Scheduled, not urgent admissions allowed	39 (36.4)	None	None
Admissions reduced	94 (87.8)	None	None
Compulsory Admissions increased	9 (8.4)	None	None
Staff meetings ^3^	72 (67.2)	None	None
Urgent Psychiatric Consultations for GeneralHospital Emergency Rooms/Other MedicalUnits	103 (96.3)	None	None
Psychiatric Consultations for Covid+ cases	23 (21.5)	*N* = 19 (33.9) C = 1 (7.1)S = 3 (9.7) *p*<.05	High rate regions^2^ = 2 (33.8) low rate regions^2^ = 5 (11 .1) *p*<.01
Psychiatric Consultations for Medical/surgicalUnits in other Hospitals^4^	Overall 58 (54.2) Only urgent 28 (26.2)	None None	High rate regions^2^ = 41 (66.1) lowrate regions^2^ = 19 (42.2) *p*<.01 High rate regions^2^ = 8 (12.9) lowrate regions^2^ = 15 (33.3) *p*<.05
Decrease of psychiatric consultations	74 (69.1)	None	High rate regions^2^ = 50 (80.6) lowrate regions^2^ = 22 (48.9) *p*<.005
Frequency of consultations according topsychiatric disorders	Delirium 35 (32.7) Mood disord 68 (63.5) Anxiety Dis 51 (47.7) Adjust Dis 37 (34.6) Personality Dis 21 (19.6) Subst use 36 (33.6) Other psyco-org dis 22 (20.5) Suicide Attempts 51 (47.7) Psychoses 56 (52.3) Complic Mourning 3 (2.8)	None	None
Inpatients violence (increase)	9 (8.4)	None	None

^1^Italy’s Geographical areas are: N=North, C=Centre, S=South and Islands

^2^group 1 ≥ 2 cases × 1000 inhabitants; group 2 < 2 cases × 1000 inhabitants;high rate regions are those included in group 1, as above; Low rate regions are those included in class 2 as above

^3^On site or in videoconference

^4^Psychiatric consultation may be required in several regions of Italy to GHPWs for other Hospitals of the same Trust

A certain reduction in the number of wards (− 13%) has been observed, mainly due to conversion into General COVID Units for positive patients, and in the number of beds available (approx.-30%), due to the need to increase the distance between patients and to set up isolation rooms. An overall reduction of admissions has been registered in 88% of GHPWs, partly due to the interruption of all scheduled admissions in approx. 64% of these units. Only 8% of GHPWs have reported an increase in compulsory admissions. On approx. One third of wards, staff meetings are no longer going ahead, whilst the vast majority of GHPWs continue to guarantee psychiatric consultations for A&E departments, and, to a lesser extent, for Medical and Surgical units. Psychiatric Consultations for COVID Units are performed in approx. One fifth of GHPWs. Mood disorders, Psychoses, Anxiety disorders and Attempted Suicides are the most frequent reasons for consultations. These data are in line with those emerging from literature with regard to psychiatric consultations for general hospitals in Italy outside a situation of emergency [30, 31]. Only 8% of Wards have registered an increased rate of violence towards self or others among inpatients. With regard to safety measures adopted (Table 5), the majority of hospitals hosting PWs have set up a “filter area” through which to access the hospital, although only 72% of these require patients to wait in these areas until the outcome of their swabs is received, prior to admission to units.

**Table 5:** Staff and user safety on General Hospital Psychiatric Wards during the Covid-19 epidemic in Italy

Items	N (%) of respondents Yes	Statistically significantdifferences accordingto geographical area^**a**^	Differences according to classesbased upon rates of Covid + cases per 1000 inhabitants on aregional basis^**b**^
“Filter” area for access to Hospital	86 (80.4)	None	None
Pts remaining in the filter areabefore swab outcomes are known	79 (73.8)	None	None
Access limitations to wards forfamily members/carers	106 (99.1)	None	None
Swabs for patients	Overall = 54 (50.5) admission+discharge = 21 (19.6) Only on admission = 28 (26.2) Only on discharge = 3 (2.8)	None None None None	None None None None
Routes dedicated to patients withsuspected infection	93 (86.9)	None	None
Voluntary Admission ofCovid+Cases to	General Covid Units^c^ = 68 (63,6) Special GHPWs^d^ = 23 (21.5) Other^e^ = 16 (14.9)	None None None	None None None
Compulsory admission of Covid+cases (managed in)	General Covid Units^c^ = 67 (62,6) Special GHPWs^d^ = 21 (19.6) Other^e^ = 19 (17.7)	*N* = 35 (60.3) C = 6 (37.5) S = 26 (78.8) *p<*.05 *N* = 11 (18.9) C = 5 (21.2) S = 6 (18.2) *N* = 12 (20.6) C = 6 (37.5) S = 1 (3.0)*	None None None
Management of agitated Covid+ pts.	Locked doors 33 (30.8) Sedation 90 (84.0) Physical restraint 42 (39.2) CCTV monitoring 31 (28.9)	None None *N* = 25 (43.1) C = 11 (68.7) S = 6 (18.1) *p*<.01 None	None None High rate reg^b^ = 30 (48.3) low rate regi^b^ = 12 (26.6) *p*<.01 None
Routine Swabs for staff membersexposed to suspected cases	48 (44.9)	None	None
Covid+ cases reported among staffmembers	30 (28.0)	*N* = 24 (41.4) C = 3 (18.7); S = 5 (15.1) *p*<.01	High rate regions^b^ = 24 (38.7) low rate regions^b^ = 6 (13.3) *p*<.01
PPD Evaluation^f^	Adequate = 17.8 Partially adequate = 52.5 Inadequate = 27.7	None None None	None None None
Staff concerns about personal safety	90 (84.1)	None	None

^a^Italy’s Geographical areas are: *N* North, *C* Centre, *S* South and Islands

^b^group 1 ≥ 2 cases × 1000 inhabitants; group 2 < 2 cases × 1000 inhabitants;high rate regions are those included in group 1, as above; Low rate regions are those included in class 2 as above

^c^General Covid Units = General Hospitals’ Intensive and Non Intensive Care Units

^d^Special GHPWs = General Hospital Psychiatric Wards specifically devoted to Covid+ patients

^e^Other = psychiatric pts. managed in isolated rooms or sections within GHPWs

^f^*PPE* Personal Protective Equipment

Visitor access to wards is prohibited in almost all GHPWs, and in the vast majority of cases (approximately 90%) specific pathways are adopted for patients with suspected COVID-19 infection. Fifty percent of GHPWs report the availability of swabs for patients; however, only 20% are able to request patient swabbing on both admission and discharge. Irrespective of whether the admission is voluntary or compulsory, about 60% of GHPWs taking part in the survey reported that COVID+ psychiatric patients with no significant behavioural disturbances may be admitted to General COVID Units; one fifth of GHPWs reported that acute patients who are unable to collaborate because of their mental condition are admitted to other GHPWs specifically set up to care for COVID+ patients, whilst approximately 15% of wards place patients in isolated areas of the ward. In the presence of agitation, measures most frequently adopted by GHPWs in controlling COVID+ patients include pharmacological sedation (approx. 85% of units) and physical restraint (about 40% of units). The latter has been adopted with significant frequency in wards located in Regions of Northern-Central Italy and areas featuring a high spread of the COVID-19 infection. Routine swabs for staff members are only available in 45% of wards, while 28% have indicated the presence of COVID positivity among workers, again more frequently in GHPWs in Northern Italy and areas with the highest rates of COVID-19 infection. In almost three quarters of wards safety measures are deemed to be inadequate or only partially adequate, while staff members in more than 80% GHPWs have expressed concerns over personal safety.

## Discussion

Before commenting on the results of this survey, a series of limitations of the study should however be acknowledged. Firstly, it should be considered that only 54% of mental health departments could be tested, and data relating to inpatient units were received for only one third of GHPWs; these samples therefore should not be taken as fully representative of all Italian services. However, the lack of any statistically significant differences with regard to geographic distribution of respondent and non-respondent MHDs and GHPWs should be taken into account. Moreover, although questionnaires were filled in by the Heads of MHDs, the possibility that data reported may not always be fully precise cannot be ruled out, even considering that for several areas the items proposed were necessarily limited in order to prevent the exercise from being excessively time consuming. Indeed, the objective difficulty of involving keyworker colleagues working on the front line in this research study should be given due consideration. However, even in the light of the abovementioned limitations, the data emerging from this survey certainly provides a fairly reliable cross-section of the reality in the context of the Italian mental health services during the acute phase of the pandemic. The severe limitation of movement for the population, patients’ fear of being contaminated, a series of national and/or regional ordinances such as those limiting intervention to urgent cases, the mandatory reduction of active staffing and respective rotas may explain, at least in part, the observed decline in activities provided by community services (decrease in number of open CMCHs, CDs and DHs, reduced access hours, restriction of activities to psychiatric visits only for severe cases, marked reduction of other activities, such as psychosocial and rehabilitative interventions, and monitoring of patients living in RFs etc...). Reports received from Italian MHDs have highlighted how care requirements for the populations concerned have been addressed through the implementation of remote contacts (phone and/or video calls), although no reliable qualitative and/or quantitative data regarding these measures are yet available at a national level; however, data emerging from this survey show that the first major trial on the application of telepsychiatry (TP) on a national scale took place in Italy during the pandemic. A recent review of studies has highlighted how mental health interventions performed remotely are as effective as those conducted face-to-face, although concerns have been raised with regards to privacy issues and the usefulness of TP in emergency situations, such as those we are currently facing [32]. However, TP is currently viewed as the most effective means for mental health services to deliver interventions under the circumstances associated with the COVID-19 pandemic [32–34], with similar solutions being adopted throughout a series of other countries [35–38]. In Italy, the measures adopted consisted in finalizing remote contacts for the purpose of providing clinical monitoring, psychological support, teaching of safety measures (i.e. social distancing and hand washing), amending pharmacological treatments as required, and collecting information on the physical health of users and caregivers, particularly fever and respiratory signs and/or symptoms. In several MHDs, individual psychotherapies were delivered using audio-visual platforms. In doing so, routine ordinary activities have been partially replaced, although the effectiveness of these substitute activities and level of satisfaction of users should be a matter of ad hoc investigations. The limited increase in reports of both voluntary and compulsory hospital admissions, and the negligible increase in aggressive behaviours in community patients seems to indicate that the shrinkage in community care does not appear to have had a substantially negative impact, at least in the short term. However, the substantial decrease in psychotherapeutic, psychosocial and rehabilitative interventions, expected to be particularly detrimental in the long run for those affected by severe mental disorders [39], viewed as representing the most vulnerable segment of the population in these circumstances, may prove challenging [34]. Moreover, the impossibility of providing new access to CMHCs may have prevented a large portion of the population exposed to the negative psychological impact of the epidemic [6] from obtaining help from the public healthcare system. The reduction of beds in GHPWs has been the price to pay for the conversion of numerous departments into Intensive COVID-19 Units, due to the dramatic lack of beds in the latter units. Likewise, the marked restriction of scheduled hospitalizations and hospital admission for only severe cases may justify the observed reduction in hospitalization rates in GHPWs. However, the potential contribution of additional factors, such as a severe limitation of access to substance users as a result of the rigid lockdown, with consequent cessation or reduction of a slatentizing effect of substances on relapses both in affective and non-affective psychotic disorders, cannot be ruled out. Moreover, with particular focus on voluntary admissions, a fear of contagion may have deterred those in need of intensive care from seeking hospitalisation. Individuals affected by mental disorders may encounter a series of obstacles in accessing health services due to a double discrimination linked to both their mental illness and to being infected by coronavirus [40]. At the current time however, it is not clear how much this feared discrimination has actually been manifested in Italy, even in the light of our data relating to the access of non-decompensated psychiatric patients to COVID-19 Units in General Hospitals. As expected from the experience of other Countries [13], the management of patients with severe psychiatric disorders in the presence of suspected or confirmed COVID-19 represents a major logistic challenge for Psychiatric Wards for which no across the board, simple solutions are currently available. Indeed, the most frequently applied solutions to this problem include the setting up of specific, separate areas within the wards or of specific Psychiatric Wards for COVID+ patients, in line with the Recommendations of the Italian Society of Psychiatry for the containment of the SARS-COV-19 virus [41]. Fortunately, the psychological burden produced by the ongoing emergency does not seem to have led to an increase in the rates of psychomotor agitation and/or violence on psychiatric wards. As anticipated, a reduction in the number of consultations in General Hospital Medical and Surgical units was registered in our survey, particularly in areas affected by a high presence of infections. The challenge of securing adequate supplies of PPE for all patients has been highlighted during this emergency [11]; however, on the basis of this survey alone, we are unable to ascertain whether a similar situation was also experienced for our inpatients. Indeed, this lack of PPE has not been confined merely to patients on psychiatric wards, but has also affected mental health workers operating in both community and hospital services. This represents a problem of both an ethical and psychological nature, particularly as the majority of MHDs have highlighted the numerous relevant concerns raised by mental health workers. Moreover, the issue likewise impinges on the health of the population, with potentially infected healthcare workers unable to access testing contributing towards spreading the infection. As reported very recently, Italy has registered a relevant number of infections among healthcare workers, with more than 12,000 subjects testing positive for COVID-19, i.e. approx. 10% of all COVID-19 cases registered in the country. This finding underlines the need to designate the protection of healthcare workers as an absolute priority for the purpose of limiting the spread of the virus [42] To summarize, the main problems identified include the management of acute, COVID+ cases in inpatient units, limitations in the monitoring of both inpatients and staff members, mainly due to the limited availability of rota swaps, and the shortage of protective devices for mental health workers. Although to date the community mental health system seems to have been successful in facing the challenges manifested, should the current restrictions to operational levels continue, particularly in the field of psychosocial interventions, it is debatable whether the system will continue to cope. Moreover, in the near future a severe strain will be placed on MHDs due to the expected increase of mental disorders as a result not only of the prolongation of home confinement and a forced, and almost radical, change of lifestyle, but above all to the added burden of an unprecedented economic crisis whereby, according to the Summer 2020 Economic Forecast, the Euro area economy will contract by 8.7% in 2020, a figure equating to 11.25% for Italy, i.e. the worst performance in the Euro area [43]. The latter may produce important consequences on mental health [44] both in terms of new cases and worsening of patients already in care, as well as an increase in suicide rates. Particular concern is raised with regard to people affected by severe mental illnesses (SMI), as such a critical event requires both resilience and coping resources that may be lacking in this group of patients, as well as a social support network [45]. Moreover, the pandemic may have further aggravated the physical health of these individuals, notoriously affected by chronic physical comorbidities, as a consequence of the acknowledged inequalities in access to physical health care and worsening of negative social determinants of health [46]. The recognition that patients with SMI represent a vulnerable population, and of the need for a more proactive approach to the monitoring of patients’ physical health and utilization of services in these critical times, may indicate the benefit of resorting to a capability approach for use in identifying barriers that prevent equitable health-care provision for these subjects [47].

In conclusion, a series of important lessons can be learnt from this pandemic. The first lesson to be learnt is that our mental health system was caught unprepared, thus indicating a mandatory need to develop a national plan to include psychiatric services for the purpose of adequately and promptly addressing a potentially similar crisis in the future. The second lesson relates to the fact that people affected by mental disorders, including those affected by SMI and their families, seem to have faced the acute phase of the epidemic in the same way as any other Italian citizen, adapting themselves with an unexpected degree of collaboration to the restrictions and the unprecedented behavioural regulations imposed by the situation. However, a deeper understanding of the impact of the crisis on those affected by mental disorders and of the coping strategies and resources adopted should be obtained by means of a specific national research programme involving random samples of service users. The third lesson focuses on remote calls and appointments, the benefits of which extend far beyond these times of crisis, representing an important means of providing assistance as a routine; this would indeed imply an impelling need to potentiate the so-called “telepsychiatry” and equip facilities homogeneously throughout the national territory with the necessary technology and training. Lastly, the fourth lesson to emerge from the post-acute phase of the pandemic is that the most severe consequences on mental health will be manifested in the near future, hand in hand with the evolution of the severe economic crisis. Indeed, one of the national priorities will necessarily be the strengthening of the workforce in mental health services, which have been more heavily affected by cuts to economic investments in recent years than other public health sectors.

## Conclusions

The pandemic has led to a relevant reduction in levels of care, which may produce a severe impact on the mental health of the population in relation to the consequences of the expected economic crisis and of the second ongoing wave of the pandemic.

## Supplementary Information

**Additional file 1.** Questionnaire on Italian Mental Health Departments

**Additional file 2.** Questionnaire on Italian General Hospital Psychiatric Wards

## Data Availability

The English versions of the questionnaires are freely available as supplementary materials.
