# Supplementary material for: Psychiatry during the Covid-19 pandemic: a survey on mental health departments in Italy
Source: BMC Psychiatry. 2020 Dec 16;20:593. doi: 10.1186/s12888-020-02997-z (PMC7739792; doi:10.1186/s12888-020-02997-z)
Supplement: Supplementary file 1 — Additional file 1. Questionnaire on Italian Mental Health Departments [file 12888_2020_2997_MOESM1_ESM.docx]

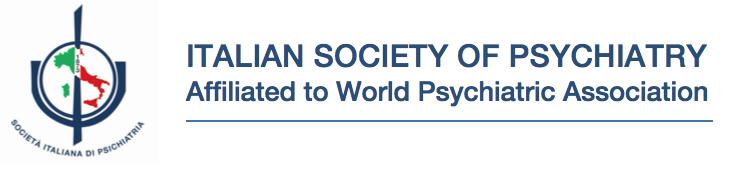


Dear Colleague,

The Italian Society of Psychiatry is collecting data about the functioning of the Italian community mental health services during the COVID-19 emergency. We would be grateful if you would answer the following questions. We thank you for your availability and cooperation. Please fill in the fields correctly, save the file and send it to [segreteria@psichiatria.it](mailto:segreteria@psichiatria.it)

| Mental Health Department |  |
| --- | --- |
| Director |  |
| Region |  |
| Reference population  (Approximate number of inhabitants) |  |

| The Department of Mental Health directed by you includes: | |
| --- | --- |
| 1) | Child Neuropsychiatry |
| 2) | Pathological Addiction Services |
| 3) | Both |

1. During the Covid-19 epidemiological emergency, as regard to the Community Mental Health Centers and any other territorial structures (if more than one):
2. they are all open
3. It has been decided a strategic closure of some of them
4. Community Mental Health Services are open:
5. Every day
6. The opening days have been reduced

3. The opening hours:

1. were reduced
2. remained unchanged

4. New and non-urgent cases are taken in care

1. Yes
2. No

5. Scheduled outpatient visits of patients already in charge are provided:

1. Yes
2. No

6. Scheduled home visits are provided:

1. Yes
2. No

7. Urgent outpatient visits are carried out

1. Yes
2. No

8. Urgent home visits (or in other places in the territory) are provided

1. Yes
2. No

9. Emergency visits and treatments are provided

1. Yes
2. No

10. Psychological visits and/or individual psychotherapies are provided

1. Yes
2. No

11. Group psychotherapies or other group activities are provided

1. Yes
2. No

12. Rehabilitative activities (any kind) are provided

1. Yes, all
2. Only some or for selected cases
3. No

13. If the Community Mental Health Services performs routine or urgent activities, are there safety procedures for patients or operators (devices, spacing, body temperature monitoring, etc.)?

1. Yes, there are formal procedures written by the head of services or other authority
2. Yes, there are only informal procedures
3. No

14. In case the ordinary interventions (visits, psychotherapies, etc..) have been interrupted, have alternative ways of contact with users been put in place?

1. Yes (specify)
2. Phone contact
3. Video contact (use of Skype, or other platforms)
4. E-mail
5. All the above, depending on the case.
6. No

15. The telephone contact or teleconsultation occurs:

1. In case of user's request
2. In case of family members request
3. Routinely performed by staff members

16. Has a specific counseling activity (by telephone or other means) been activated by the Mental Health Department for the general population in relation to the epidemiological emergency?

1. Yes
2. No, but it is being carried out by other agencies or private services ( Italian Psychiatric Association, Italian Association of psychologists association of psychologists, etc.).
3. No

17. Has a specific counseling activity (by telephone or other means) been activated by the Mental Health Department for health care professionals in relation to the epidemiological emergency?

1. Yes
2. No, but it is being carried out by other agencies or private services (as above)
3. No

18. Do team meetings continue in Community Mental Health Services?

1. Yes, in a videoconferencing mode
2. Yes, in presence respecting safety measures (distancing, use of facial mask etc..)
3. No

19. Are telephone interview/consulting activities recorded in the electronic system?

1. Yes, with a separate new specific code
2. Yes, with the usual telephone contact code
3. No

20. Does the Community Mental Health Service normally carry out psychiatric consultations for local General Hospitals?

1. Yes
2. No

21. If so, do hospital consultations continue to be provided during the epidemiological emergency?

1. Yes (Only urgent ones)
2. No

22. Is the administration of oral medication to patients provided when deemed necessary?

1. Yes, even at home if it is the case
2. Yes, only at the Community Mental Health Service
3. No

23. Are injections of Long Acting antipsychotics (LAI) provided?

1. Yes, even at home if this is the case
2. Yes, but only at the Community Mental Health Service
3. No, temporarily LAIs are replaced by oral therapy

24. Have territorial Day Hospital (DH) activities (if any) been suspended?

1. Yes
2. No

25. The activities of the Day Centers (DC) (if any) have been suspended.

1. Yes
2. No

26. Have patient monitoring procedures at Residential Facilities been suspended (if they involve access by Community Mental Health Centers operators at the facilities)?

1. Yes
2. No

27. Have new entries in Residential Facilities been suspended or reduced?

1. Yes
2. No

28. Have the scheduled home returns of patients admitted to Residential Facilities been suspended?

1. Yes
2. No

29. Has the scheduled clinical monitoring of offending psychiatric patients been suspended?

1. Yes
2. Yes, only after communicating it to the competent Judge
3. No

30. Does the Mental Health Department usually provide psychiatric counseling in prison?

1. Yes
2. No

31. If so, will prison psychiatric counseling continue to be provided during the present emergency?

1. Yes
2. Yes, but only for urgent cases
3. No

32. Addiction Services (AS) are open:

1. Every day
2. The opening days have been reduced

33. The opening hours of AS:

1. Has been reduced
2. They remained unchanged

34. Are there any Regional Recommendations or Guidelines that regulate the suspension or reduction of the above-mentioned activities in Community Mental Health Centres, Day Centers, Day Hospitals, Residential facilities?

1. Yes
2. No
3. Have the community services been equipped with Personal Protective Equipment (PPE)?
4. Yes (specify)
5. Remote thermometers
6. Surgical masks
7. FFP2/3 masks
8. Gloves
9. Safety glasses
10. Disposable protective gowns
11. No
12. The devices provided are, in your opinion, overall:
13. Adequate in terms of type and quantity
14. Partially adequate
15. Inadequate
16. Have there been cases of fiduciary quarantine among staff members?
    1. Yes
    2. No
17. Have there been cases of Covid related disease among staff members?
    1. Yes
    2. No
18. Have there been any cases of Covid related disease among patients in charge of community services?
    1. Yes
    2. No
19. Have there been any cases of Covid related disease among patients placed in residential facilities?
    1. Yes
    2. No
20. Have staff members expressed concerns about anti-Covid-19 security measures adopted in the workplace (including off-site activities and emergency treatments)?
    1. Yes
    2. No
21. If yes, how many have expressed fears?
    1. Most of them
    2. Only a part
    3. Only single cases
22. Has there been an increase of admission to hospital psychiatric services in one or more areas of the Mental Health Department since the emergency began?
    1. Yes
    2. No
23. If so, to what extent?
    1. A slight increase (approximately within 10%)
    2. A moderate increase (roughly over 10% but less than 30%)
    3. A marked increase (over 30%)
24. Has there been an increase in emergency treatment in one or more areas of the Mental Health Department since the emergency began?
    1. Yes
    2. No
25. If yes, to what extent?
    1. A slight increase (approximately within 10%)
    2. A moderate increase (roughly over 10% but less than 30%)
    3. A marked increase (over 30%)
26. Has there been an increase in aggression/violence cases in one or more areas of the Mental Health Department since the emergency began?
    1. Yes
    2. No
27. If yes, to what extent?
    1. Isolated cases, not serious (increased intra-family conflict, with verbal aggression, threats)
    2. Isolated cases, more worrying (physical violence)
    3. Several cases, not serious (increased intra-family conflict, with verbal aggression, threats)
    4. Several cases, serious (physical violence)
