# Supplementary material for: Psychiatry during the Covid-19 pandemic: a survey on mental health departments in Italy
Source: BMC Psychiatry. 2020 Dec 16;20:593. doi: 10.1186/s12888-020-02997-z (PMC7739792; doi:10.1186/s12888-020-02997-z)
Supplement: Supplementary file 2 — Additional file 2. Questionnaire on Italian General Hospital Psychiatric Wards [file 12888_2020_2997_MOESM2_ESM.docx]

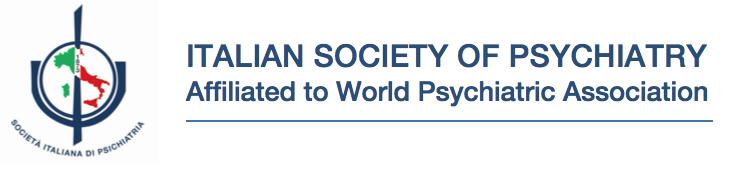


Dear Colleague,

The Italian Society of Psychiatry is collecting data about functioning of the Italian General Hospital Psychiatric Wards (GHPW) during the COVID-19 emergency. We would be grateful if you would answer the following questions. Thank you for your availability and cooperation. Please fill in the fields correctly, save the file and send it to [segreteria@psichiatria.it](mailto:segreteria@psichiatria.it)

| Psychiatric ward |  |
| --- | --- |
| Director |  |
| Region |  |
| Number of hospital beds |  |
| Area |  |

1. Considering the GHPW of your area, please let’s know if:
   - 1. They are all open
     2. a strategic closure of some of them was implemented
2. In the open ones, a reduction of beds has been implemented?
   - 1. Yes
     2. No
3. In the open ones, has the visitors' access mode been changed? (i.e. Visiting hours, number of relatives per patient, pre-triage by questionnaire, suspension of visits)
   - 1. Yes
     2. No
4. The outpatient activity of the GHPW has been suspended?
   - 1. Yes
     2. No
     3. Outpatient activities are not provided
5. In the case of suspended outpatient activity, contacts with the patients are maintained by means of :
   - 1. telephone
     2. video-calls ( eg Skype or other platforms)
     3. E-mail
     4. all the precedents according to the case
6. The contacts mentioned in the previous point are mantained:
   1. Mainly in scheduled mode
   2. Mainly on request of the patient
7. Are scheduled hospitalizations provided?
   1. Yes
   2. No
8. Has there been an increase of compulsory admissions?
   1. Yes
   2. No
9. Have you observed a overall reduction in admissions?
   1. Yes
   2. No
10. Are the team meetings continuing?
    1. Yes
    2. No
11. Do psychosocial activities of any kind continue, if provided in the ward?
    1. Yes
    2. Yes, only some activities or for selected cases
    3. No
12. The GHPW has been equipped with personal protection devices (indicate all those supplied):
    1. Remote thermometers
    2. Surgical masks
    3. FFP2/3 masks
    4. Protective Glasses
    5. Overcoats
13. In your opinion the above mentioned devices are on the average:
    1. Adequate in terms of type and quantity
    2. Partially adequate
    3. Inadequate
14. Have there been cases of Covid 19 positivity among staff members?
    1. Yes
    2. No
15. Are swabs routinely provided for staff members in case of contact with positive patients/family members?
    1. Yes
    2. No
    3. If yes, when……… (how long it takes before the swab is provided)
16. Have staff members expressed concerns about the anti-Covid security measures provided?
    - 1. Yes
      2. No
17. Is there a filter area for new admissions outside the Psychiatric Ward (in the Emergency Room or in a tent in a outside area)?

a) Yes

b) No

1. A psychiatric patient who is suspected for COVID or is COVID positive follows the same paths as non-psychiatric patients?
   1. Yes
   2. No
2. While waiting for the response to the first swab, does the patient remain in the filter area?
   1. Yes
   2. No
3. Have safety measures been adopted for patients at the entrance? (Isolation room or other)
   1. Yes
   2. No
4. In the case of a patient who is isolated and agitated, what options do you adopt (indicate all those used) ?
   1. Closed-doors
   2. Pharmaceutical tranquilization
   3. Physical restraint
   4. Camera
5. Are swabs for Sars-Cov-2 provided for patients on admission and/or discharge?
   1. Yes, both on admission and discharge
   2. No
   3. Only on admission
   4. Only on discharge
6. Has there been an increase in episodes of aggression/violence in the PW during the current pandemic?
   1. Yes
   2. No
7. Covid-19 positive patients who are admitted according to a compulsory treatment order:
   1. Undergo the same Care Paths as any other patient
   2. Are transferred to a Psychiatric Ward specifically dedicated to Covid-19 positive patients or to a dedicated area in the PW
8. Covid-19 positive patients who are admitted on a voluntary basis:
   1. Undergo the same Care Paths as other patients
   2. Are transferred to a Psychiatric Ward specifically dedicated to Covid-19 positive patients or to a dedicated area in the PW
9. Do You receive referrals from other medical Units for Covid-19 positive patients due to a lack of Consent to treatment:
   1. Yes
   2. No
10. Are urgent psychiatric consultations provided for the Emergency or other departments/units of the Hospital?
    1. Yes
    2. No
11. Do your PW provide psychiatric consultation for departments/ units located in other Hospitals of your area?
    1. Yes
    2. Yes, but only for urgent cases
    3. No
12. Has there been a change in the amount of requests for psychiatric consultations received from the medical and surgical departments?
    1. Yes, they are increased
    2. No
    3. Yes, they are decreased
13. For which psychiatric disorders psychiatric consultation are mostly requested? (multiple answers are possible)
    - 1. Mood Disorders
      2. Anxiety disorders
      3. Stress Disorders (PTSD, Adjustment Disorder)
      4. Personality Disorders
      5. Substance use/abuse disorders
      6. Delirium
      7. Psychorganic disorders
      8. Attempted suicide
      9. Psychotic Disorders
      10. Complicated mourning
